# Oxidative Stress in Inflammatory Bowel Disease: From Redox Dysregulation to Translational Targeting

**DOI:** 10.3390/antiox15070894

**Published:** 2026-07-20

**Authors:** Alexandra Laura Mederle, Ana Lascu, Andrei Raul Manzur, Alexandru Caraba, Raluca Elisabeta Staicu, Lavinia Noveanu, Oana Maria Aburel

**Affiliations:** 1 Doctoral School Medicine-Pharmacy, “Victor Babes” University of Medicine and Pharmacy Timisoara, Eftimie Murgu Square No. 2, 300041 Timisoara, Romania; alexandra.mederle@umft.ro (A.L.M.); raluca.staicu@umft.ro (R.E.S.); 2 Department of Microbiology, Discipline of Dermatology, “Victor Babes” University of Medicine and Pharmacy, Eftimie Murgu Square 2, 300041 Timisoara, Romania; 3 Department of Functional Sciences, Discipline of Physiopathology, Faculty of Medicine, “Victor Babes” University of Medicine and Pharmacy, Eftimie Murgu Sq. No. 2, 300041 Timisoara, Romania; noveanu@umft.ro (L.N.); oanaduicu@umft.ro (O.M.A.); 4 Centre for Translational Research and Systems Medicine, “Victor Babeș” University of Medicine and Pharmacy of Timișoara, 300041 Timisoara, Romania; 5 Institute for Cardiovascular Diseases of Timisoara, Clinic for Cardiovascular Surgery, 300310 Timisoara, Romania; 6 Department of Internal Medicine, Diabetes, Nutrition and Metabolic Diseases, “Victor Babeș” University of Medicine and Pharmacy, 300041 Timisoara, Romania; caraba.alexandru@umft.ro; 7 Railway Clinical Hospital Timișoara, 300173 Timisoara, Romania; 8 Institute for Cardiovascular Diseases, Department of Anesthesiology and Intensive Care, "Victor Babeș" University of Medicine and Pharmacy Timișoara, Gheorghe Adam St. 13A, 300310 Timișoara, Romania

**Keywords:** inflammatory bowel disease, oxidative stress, redox biomarkers, Crohn’s disease, ulcerative colitis, mitochondrial dysfunction, ferroptosis, NF-κB, microbiome, precision medicine, antioxidant therapy

## Abstract

Oxidative stress has emerged as an important component of the complex pathophysiology of inflammatory bowel disease (IBD), where increasing evidence suggests an interaction between redox imbalance, immune activation, epithelial dysfunction, and chronic intestinal inflammation. This structured narrative review critically synthesizes current evidence regarding the biological basis of oxidative stress in IBD, with emphasis on cellular and molecular mechanisms, oxidative biomarkers, therapeutic modulation of redox pathways, and their translational relevance. Current evidence indicates that oxidative stress is associated with immune-cell activation, mitochondrial dysfunction, impairment of epithelial homeostasis, and dysregulation of redox-sensitive signaling pathways. Biomarkers including nitric oxide metabolites, malondialdehyde, myeloperoxidase, total antioxidant capacity, serum thiols, and antioxidant enzymes have demonstrated associations with inflammatory activity, while anti-inflammatory, antioxidant, and dietary interventions have been reported to modulate oxidative biomarkers in selected clinical studies. However, substantial methodological heterogeneity, variability in analytical techniques, and limited prospective validation currently restrict their routine clinical application. Moreover, many mechanistic pathways have been characterized predominantly in experimental models, highlighting the need to distinguish biological plausibility from evidence supporting clinical implementation. Overall, oxidative stress represents a promising area of investigation that may contribute to a better understanding of IBD biology and support future biomarker-guided and precision medicine approaches. Nevertheless, further standardized translational and longitudinal clinical studies are required before oxidative biomarkers and redox-targeted strategies can be integrated into routine patient care.

## 1. Introduction

Inflammatory bowel disease (IBD), encompassing Crohn’s disease (CD) and ulcerative colitis (UC), comprises chronic, relapsing inflammatory disorders of the gastrointestinal tract that arise from a complex interaction between genetic susceptibility, immune dysregulation, environmental exposures, intestinal microbiota, and epithelial barrier dysfunction [1,2,3]. Although substantial advances have been achieved in understanding the immunopathogenesis of IBD and in developing biologic and small-molecule therapies, the mechanisms responsible for disease initiation, progression, and heterogeneity remain only partially understood.

Among the biological processes implicated in IBD, oxidative stress has emerged as an important component of the inflammatory microenvironment. Oxidative stress develops when the production of reactive oxygen species (ROS) and reactive nitrogen species (RNS) exceeds the capacity of endogenous antioxidant defense systems, leading to disruption of cellular redox homeostasis [4,5,6]. Experimental and clinical studies have associated redox imbalance with epithelial barrier dysfunction, immune-cell activation, mitochondrial injury, and amplification of inflammatory signaling in both CD and UC. However, the extent to which oxidative stress represents a consequence of chronic inflammation, a contributor to disease progression, or both remains an area of active investigation [4,5,6].

Growing interest in redox biology has also stimulated research into oxidative stress-related biomarkers and therapeutic strategies. Biomarkers such as malondialdehyde (MDA), myeloperoxidase (MPO), nitric oxide metabolites, total antioxidant capacity (TAC), serum thiols, and antioxidant enzymes have been investigated as potential indicators of disease activity and treatment response, while antioxidant-based interventions have been explored as adjunctive therapeutic approaches. Despite these advances, translation into routine clinical practice remains limited because of methodological heterogeneity, lack of standardized assays, and inconsistent validation across patient populations [7,8,9,10].

Several reviews have summarized individual aspects of oxidative stress in IBD; however, the rapid expansion of research in molecular signaling, mitochondrial biology, host–microbiome interactions, ferroptosis, and multi-omics approaches warrants an updated integrative synthesis. In addition, increasing emphasis on translational medicine has highlighted the need to critically evaluate the clinical applicability of oxidative biomarkers and redox-targeted interventions while distinguishing established evidence from emerging experimental concepts.

Accordingly, this structured narrative review provides an updated overview of oxidative stress in inflammatory bowel disease by integrating current evidence on its biological basis, cellular and molecular mechanisms, oxidative biomarkers, therapeutic targeting, and translational implications. Particular emphasis is placed on critically appraising the strength of the available evidence, distinguishing findings derived from clinical studies and experimental models, and identifying current limitations and future directions for the integration of redox biology into precision medicine strategies for IBD.

## 2. Materials and Methods

This review was conducted as a structured narrative review to synthesize current evidence regarding the role of oxidative stress in inflammatory bowel disease, with particular emphasis on its biological basis, molecular mechanisms, oxidative biomarkers, therapeutic modulation, and translational implications. The review was designed to integrate mechanistic, experimental, and clinical evidence rather than to perform a systematic review or quantitative meta-analysis.

A comprehensive literature search was performed using the PubMed/MEDLINE, Scopus, Embase, Web of Science, and Google Scholar databases. Publications from January 1986 through December 2024 were considered. The search strategy combined Medical Subject Headings (MeSH) and free-text keywords, including “oxidative stress,” “reactive oxygen species,” “reactive nitrogen species,” “inflammatory bowel disease,” “Crohn’s disease,” “ulcerative colitis,” “redox biomarkers,” “mitochondrial dysfunction,” “NF-κB,” “Nrf2,” “ferroptosis,” and “antioxidant therapy.” Reference lists of eligible articles and relevant review papers were also manually screened to identify additional studies.

Articles published in English were considered for inclusion. Priority was given to original clinical investigations, observational studies, randomized and non-randomized interventional studies, translational research, systematic reviews, and meta-analyses directly addressing oxidative stress or redox biology in Crohn’s disease or ulcerative colitis. Experimental studies involving animal models or intestinal epithelial cell systems were included when they provided mechanistic insights relevant to human IBD. Studies focusing exclusively on diseases unrelated to IBD or lacking relevance to oxidative stress pathways were excluded.

The retrieved literature was independently screened by two authors, with disagreements resolved through discussion and consensus among the review team. Because this was a narrative review, no formal risk-of-bias assessment or quantitative evidence synthesis was performed.

The selected literature was synthesized thematically according to the principal domains of oxidative stress research in IBD, including the biological basis of redox imbalance, cellular and molecular mechanisms, oxidative biomarkers, therapeutic strategies, translational challenges, and future research directions. Throughout the manuscript, an effort was made to distinguish evidence derived from human clinical studies, animal models, and in vitro investigations in order to reflect the relative strength of the available evidence and to avoid overinterpretation of experimental findings.

## 3. Oxidative Stress in IBD: Biological Basis

Oxidative stress has emerged as an important component of the complex pathophysiology of IBD. During intestinal inflammation, activated neutrophils, macrophages, monocytes, and intestinal epithelial cells produce increased amounts of ROS and RNS as part of the innate immune response. Under physiological conditions, these reactive species contribute to antimicrobial defense, intracellular signaling, and maintenance of mucosal homeostasis. However, sustained inflammatory activity may disrupt the balance between oxidant production and endogenous antioxidant defenses, resulting in oxidative stress and redox imbalance [4,5,6].

**Figure 1:**
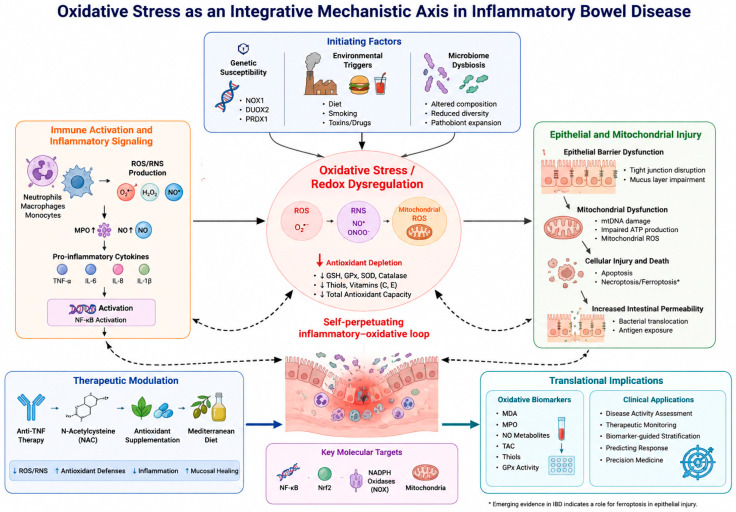
Integrative overview of oxidative stress in IBD. Genetic susceptibility, environmental factors, gut microbiota dysbiosis, and chronic immune activation promote excessive ROS and RNS production, resulting in redox imbalance. Oxidative stress is associated with epithelial barrier dysfunction, mitochondrial impairment, inflammatory signaling, and depletion of endogenous antioxidant defenses. These interconnected processes may contribute to intestinal inflammation while providing opportunities for biomarker development and redox-targeted therapeutic strategies. ↑ increased; ↓ decreased.

The intestinal mucosa is particularly susceptible to oxidative injury because it is continuously exposed to luminal microorganisms, dietary antigens, and environmental stimuli while maintaining a highly active epithelial barrier. Persistent immune activation may further increase oxidative burden through ongoing inflammatory cell recruitment and cytokine production, establishing a bidirectional interaction in which inflammation promotes oxidative stress, while oxidative stress may further amplify inflammatory processes [4,5,6]. Although this relationship is supported by experimental and clinical observations, the relative contribution of oxidative stress to disease initiation and progression remains incompletely understood. The principal biological processes linking oxidative stress with chronic intestinal inflammation are summarized in Figure 1.

Experimental and clinical studies have associated oxidative stress with several pathological features of IBD, including epithelial barrier dysfunction, mitochondrial impairment, depletion of endogenous antioxidant defenses, and disruption of intestinal homeostasis [4,5,6,7,8,11]. Oxidative modification of lipids, proteins, and nucleic acids has been reported in patients with both CD and UC, supporting the concept that redox imbalance accompanies active intestinal inflammation. However, much of the currently available evidence is derived from observational studies and mechanistic investigations, and therefore should be interpreted primarily as demonstrating biological association rather than definitive causality.

Collectively, the available evidence suggests that oxidative stress represents an important biological component of the inflammatory microenvironment in IBD. Its interaction with immune activation, epithelial integrity, and cellular metabolism has generated considerable interest in understanding the molecular pathways involved and in evaluating their potential clinical relevance. These cellular and molecular mechanisms are discussed in the following chapter.

## 4. Cellular and Molecular Mechanisms

Oxidative stress in IBD reflects a complex interaction between immune-cell activation, epithelial dysfunction, mitochondrial impairment, and redox-sensitive intracellular signaling. Rather than representing a single pathogenic pathway, oxidative stress encompasses multiple interconnected biological processes that may contribute to intestinal inflammation and mucosal injury through distinct but overlapping mechanisms [4,5,6].

One of the earliest recognized mechanisms involves excessive production of ROS by activated innate immune cells. Neutrophils, macrophages, and monocytes infiltrating the intestinal mucosa generate ROS as part of the physiological antimicrobial response. During chronic inflammation, however, persistent activation of these cells may result in sustained oxidative activity, increasing the likelihood of epithelial injury and disruption of mucosal homeostasis [4,5,6]. Early clinical studies by Curran et al. demonstrated enhanced neutrophil-derived superoxide production in patients with CD, while Baldassano et al. subsequently reported increased oxidative burst activity in circulating monocytes, supporting the presence of altered innate immune-cell redox responses in IBD [11,12]. Although these studies provided important mechanistic insights, they were relatively small and primarily demonstrated associations between inflammatory activity and oxidative responses rather than establishing causality.

In addition to ROS, excessive generation of RNS has also been implicated in chronic intestinal inflammation. Increased activity of inducible nitric oxide synthase and elevated concentrations of nitrite and nitrate metabolites have been reported in patients with active CD and UC, suggesting enhanced nitrosative stress during intestinal inflammation [13,14,15]. Experimental evidence further indicates that interactions between ROS and RNS may promote formation of highly reactive intermediates capable of inducing lipid peroxidation, protein oxidation, DNA damage, and disruption of epithelial integrity [14,16,17]. Nevertheless, considerable variability exists among studies regarding biological sample type, analytical methodology, and disease activity assessment, limiting direct comparison of findings.

Oxidative injury is further influenced by impairment of endogenous antioxidant defense systems. Reduced activity of enzymatic antioxidants, including GPx, SOD, and CAT, together with depletion of non-enzymatic antioxidants such as thiol-containing compounds, has been described in patients with active IBD [7,8,18,19,20]. These alterations may reduce the capacity of intestinal tissues to neutralize ROS and RNS, thereby favoring persistent redox imbalance. However, antioxidant depletion may also reflect the inflammatory state itself, and current evidence does not clearly establish whether these changes precede disease exacerbation or occur predominantly as a consequence of ongoing inflammation.

Recent investigations have expanded the understanding of oxidative stress beyond direct oxidant production to include alterations in intracellular signaling and cellular metabolism. Redox-sensitive pathways involving NF-κB, Nrf2, NOX1, DUOX2, and SMOX have been associated with regulation of inflammatory responses, antioxidant defenses, epithelial integrity, and cellular adaptation to oxidative stress [4,6,21,22,23,24]. Experimental studies have also highlighted the contribution of mitochondrial dysfunction, characterized by impaired oxidative phosphorylation, altered ATP production, and increased mitochondrial ROS generation, to epithelial injury and chronic inflammation [18,19]. In parallel, emerging evidence suggests that ferroptosis, an iron-dependent form of regulated cell death associated with lipid peroxidation and GPX4 dysfunction, may represent an additional mechanism contributing to intestinal epithelial damage, although current evidence remains largely experimental [24].

The interaction between oxidative stress and the intestinal microbiota has also received increasing attention. Multi-omics analyses and experimental studies suggest that oxidative pathways may influence microbial composition and epithelial responses, while alterations in the microbiota may further modulate oxidative signaling and inflammatory activity [4,5,6,23]. These bidirectional interactions are increasingly recognized as components of the complex biological network underlying chronic intestinal inflammation, although their precise contribution to disease heterogeneity requires further investigation.

Representative studies investigating the principal cellular and molecular mechanisms associated with oxidative stress in IBD are summarized in Table 1.

Collectively, the available evidence supports the concept that oxidative stress is closely integrated with immune activation, epithelial dysfunction, and cellular signaling in IBD. However, the strength of evidence varies substantially across mechanisms. While several pathways are supported by observations in human tissues and clinical studies, others rely predominantly on animal models or in vitro investigations. Continued translational research integrating molecular, cellular, and clinical data will be essential to clarify the relative contribution of these mechanisms to disease pathogenesis and to identify those with the greatest therapeutic potential.

## 5. Oxidative Biomarkers

The growing interest in oxidative stress as a component of IBD pathophysiology has stimulated the investigation of numerous oxidative biomarkers as potential indicators of inflammatory activity and therapeutic response. Biomarkers reflecting oxidative damage, antioxidant depletion, and redox-sensitive signaling have been evaluated in serum, plasma, intestinal tissue, fecal samples, and, less frequently, saliva. Although many studies have demonstrated associations between these biomarkers and disease activity, their clinical applicability remains limited by methodological heterogeneity and insufficient validation [7,8].

Among the most extensively investigated biomarkers are NO metabolites, MDA, MPO, TAC, serum thiols, and antioxidant enzymes including GPx, SOD, and CAT. Collectively, these biomarkers reflect different aspects of oxidative biology, ranging from lipid peroxidation and neutrophil activation to impairment of endogenous antioxidant defenses. Rather than representing interchangeable indicators, each biomarker provides information on a specific component of redox homeostasis and therefore should be interpreted within its biological context.

The principal oxidative biomarkers investigated in IBD and their proposed translational relevance are summarized in Table 2.

NO metabolites were among the earliest oxidative biomarkers investigated in IBD. Increased concentrations of nitrite and nitrate have been reported in patients with active CD and UC, suggesting enhanced nitrosative stress during intestinal inflammation [13,14,15,16,28]. Similarly, elevated concentrations of MDA and MPO have consistently been associated with active disease, reflecting increased lipid peroxidation and neutrophil activation, respectively [4,6,26,28]. In contrast, TAC, serum thiols, GPx, SOD, and CAT are generally reduced during active inflammation, indicating impaired antioxidant defenses [7,8,18,19,20,28]. Although these observations have been reproduced across multiple studies, considerable variation exists regarding assay methodology, biological matrices, and patient populations, limiting direct comparison between investigations.

Several studies have also explored the relationship between oxidative biomarkers and disease activity. Higher concentrations of oxidative stress markers have frequently been associated with active intestinal inflammation, whereas partial restoration of antioxidant capacity has been reported following effective anti-inflammatory treatment [7,8,26,27,28]. However, these associations have predominantly been demonstrated in observational or exploratory studies, and relatively few investigations have evaluated their ability to predict clinically relevant outcomes such as relapse, sustained remission, hospitalization, or long-term therapeutic response.

Despite their biological relevance, oxidative biomarkers have not demonstrated sufficient evidence to replace or complement established markers currently used in routine clinical practice [9,10]. Unlike CRP and fecal calprotectin, which have well-established roles in monitoring inflammatory activity and are incorporated into contemporary clinical management algorithms, oxidative biomarkers remain largely investigational [10]. At present, there is limited evidence supporting validated diagnostic thresholds, sensitivity, specificity, or AUC values for most oxidative markers in IBD [9,10]. Furthermore, no individual biomarker has consistently demonstrated independent predictive value beyond established inflammatory markers across diverse patient populations [9,20,21]. Consequently, current evidence does not support their routine clinical implementation as standalone diagnostic or prognostic tools.

**Figure 2:**
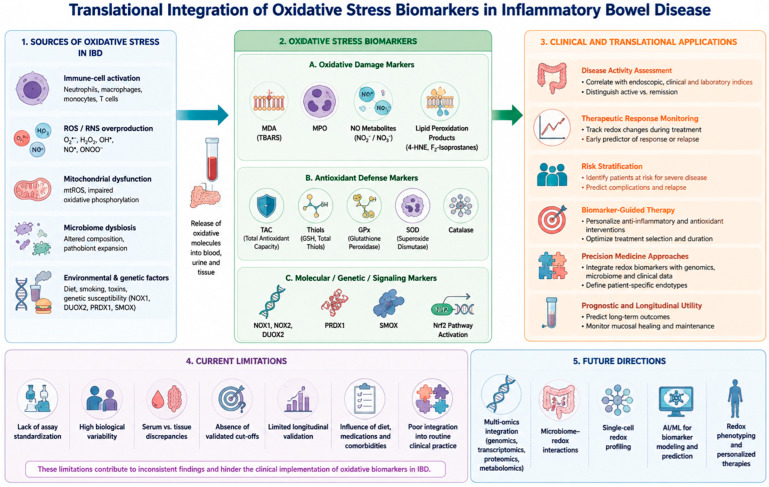
Potential clinical integration of oxidative stress biomarkers in IBD. Oxidative biomarkers may complement conventional inflammatory assessment by providing information on redox imbalance, antioxidant status, and oxidative tissue injury. Potential applications include disease activity assessment, therapeutic monitoring, and biomarker-guided patient stratification. Current limitations include methodological heterogeneity, limited standardization, and insufficient clinical validation.

The potential integration of oxidative biomarkers into future clinical practice is illustrated in Figure 2.

Nevertheless, oxidative biomarkers continue to provide important mechanistic insights into the biological processes underlying intestinal inflammation. Rather than competing with conventional inflammatory markers, future research may explore their role as complementary indicators within multimarker approaches integrating clinical, biochemical, molecular, and microbiome-derived information. Before such strategies can be implemented, larger prospective studies using standardized analytical methodologies and clinically meaningful outcome measures will be required to determine whether oxidative biomarkers provide incremental value beyond existing diagnostic and monitoring tools.

## 6. Therapeutic Targeting of Redox Pathways

The recognition of oxidative stress as an important component of the inflammatory microenvironment in IBD has stimulated interest in therapeutic strategies capable of modulating redox imbalance. Although currently approved therapies primarily target inflammatory pathways rather than oxidative stress itself, several pharmacological and nutritional interventions have been associated with changes in oxidative biomarkers, suggesting that redox homeostasis may be modified as inflammation is controlled [4,5,6].

Among the most extensively investigated therapeutic approaches are TNF-α inhibitors. Clinical studies have reported reductions in oxidative stress markers following treatment with infliximab or adalimumab in patients with active CD, accompanied by improvements in clinical disease activity [25]. These findings suggest that suppression of intestinal inflammation may partially restore redox balance. However, the available evidence does not clearly establish whether these changes represent a direct effect on oxidative pathways or occur secondary to reduced inflammatory activity. In addition, most studies have involved relatively small patient populations and have relied on surrogate biomarker outcomes rather than long-term clinical endpoints.

Antioxidant supplementation has also been explored as a potential adjunctive strategy. NAC, a precursor of glutathione synthesis, has been investigated for its capacity to replenish intracellular antioxidant defenses and attenuate oxidative injury. In a randomized crossover pilot study, administration of NAC during thiopurine therapy was associated with favorable changes in selected oxidative biomarkers, including MDA and MPO [26]. Nevertheless, these findings should be interpreted cautiously because they originate from a pilot study with limited statistical power and were not designed to demonstrate improvements in long-term clinical outcomes.

Dietary interventions represent another area of increasing interest. A randomized clinical trial evaluating a Mediterranean-style dietary intervention in patients with UC demonstrated improvements in TAC together with reductions in inflammatory markers [27]. Although these observations support the concept that nutritional strategies may influence oxidative balance, it remains uncertain whether modulation of oxidative stress directly contributes to the observed clinical benefits or primarily reflects reduced intestinal inflammation. Additional controlled studies are needed to clarify these relationships.

Experimental investigations have also evaluated a variety of antioxidant compounds and redox-modulating strategies targeting mitochondrial function, ROS generation, and intracellular signaling pathways [6,19]. While many of these approaches have demonstrated promising biological effects in preclinical models, relatively few have progressed to adequately powered clinical trials. Consequently, evidence supporting their routine clinical application in patients with IBD remains limited.

Overall, current evidence suggests that oxidative stress may represent a therapeutically relevant biological pathway in IBD. However, most available data are derived from exploratory clinical studies or experimental investigations, and there is currently insufficient evidence to recommend redox-targeted interventions as standalone therapeutic strategies. Future studies should prioritize well-designed randomized controlled trials incorporating standardized oxidative biomarkers together with clinically meaningful outcomes, including endoscopic healing, sustained remission, and long-term disease control.

## 7. Translational Challenges and Clinical Perspectives

Despite substantial advances in understanding the role of oxidative stress in IBD, the translation of these findings into routine clinical practice remains limited. Although numerous oxidative biomarkers have demonstrated associations with inflammatory activity, relatively few have undergone rigorous clinical validation. Consequently, their current role is primarily investigational, and they should not be considered substitutes for established clinical, biochemical, endoscopic, or histological assessments [7,8].

One of the principal challenges is the considerable methodological heterogeneity among published studies. Differences in patient selection, disease phenotype, disease activity, biological sample type, analytical methodology, and biomarker quantification have limited direct comparison between investigations [7,8]. Furthermore, oxidative biomarkers have been measured in serum, plasma, intestinal tissue, fecal samples, and other biological matrices, making interpretation and standardization particularly challenging.

Another important limitation is the lack of standardized analytical protocols and clinically validated diagnostic thresholds. Unlike established biomarkers such as CRP and fecal calprotectin, oxidative biomarkers currently lack universally accepted cut-off values and have not consistently demonstrated sufficient sensitivity, specificity, or reproducibility for routine clinical implementation [10,29]. Consequently, their incremental diagnostic or prognostic value beyond conventional inflammatory markers remains uncertain [7,8,9].

Interpretation of oxidative biomarkers is further complicated by the multifactorial nature of redox biology. Oxidative stress may be influenced not only by intestinal inflammation but also by diet, smoking status, medication use, metabolic disorders, microbiota composition, and other systemic inflammatory conditions. In addition, circulating nitrate and nitrite concentrations may be substantially affected by dietary nitrate intake and systemic nitrate metabolism, further complicating the interpretation of nitrosative stress biomarkers [23,30,31,32]. These factors may contribute to interindividual variability and reduce biomarker specificity, particularly when measurements are performed outside standardized clinical settings.

An additional challenge concerns the interpretation of mechanistic evidence. Many pathways implicated in oxidative stress, including mitochondrial dysfunction, ferroptosis, and several redox-sensitive signaling pathways, have been characterized primarily in experimental models. Although these investigations provide valuable biological insight, their direct clinical relevance remains incompletely established. Future translational research should continue to distinguish findings derived from human studies from those obtained in animal or in vitro models to facilitate accurate interpretation of the available evidence.

Taken together, the current literature supports oxidative stress as an important biological component of IBD, but several obstacles must be addressed before oxidative biomarkers or redox-targeted strategies can be incorporated into routine clinical practice. Standardization of laboratory methodologies, prospective multicenter validation studies, integration with established clinical and endoscopic indices, and evaluation of clinically meaningful outcomes will be essential to determine whether oxidative biomarkers provide additional value within future precision medicine approaches.

## 8. Future Directions

Advancing the clinical application of oxidative stress research in IBD will require a transition from descriptive biomarker studies toward integrated translational investigations capable of demonstrating clinical utility. Future research should prioritize well-designed prospective multicenter studies employing standardized analytical methodologies, harmonized patient stratification, and clinically relevant outcome measures to improve the reproducibility and comparability of oxidative biomarker research.

Rather than evaluating individual oxidative biomarkers in isolation, future studies may benefit from integrating redox-related parameters with established inflammatory markers, endoscopic findings, histopathological assessment, and molecular profiling. Such multimodal approaches may improve disease phenotyping, facilitate patient stratification, and provide a more comprehensive understanding of disease heterogeneity than any single biomarker alone.

Recent advances in genomics, transcriptomics, metabolomics, and microbiome research have further expanded opportunities to investigate oxidative stress within a broader systems biology framework. Integration of multi-omics data with redox biology may help identify distinct molecular endotypes, improve understanding of disease mechanisms, and support the development of more individualized therapeutic strategies [24].

Continued investigation of mitochondrial biology, redox-sensitive signaling pathways, and regulated cell death mechanisms, including ferroptosis, may further clarify their contribution to intestinal inflammation and identify novel therapeutic targets. However, translation of these experimental findings into clinical practice will require rigorous validation in human studies before their clinical significance can be fully established [19,24].

Ultimately, the greatest potential of oxidative stress research in IBD may lie not in replacing existing diagnostic or therapeutic approaches but in complementing current clinical practice through improved biological characterization of disease, more precise patient stratification, and the identification of novel therapeutic opportunities. Achieving these goals will require close collaboration between basic scientists, translational investigators, and clinicians to ensure that future discoveries are supported by robust clinical evidence and can be effectively integrated into precision medicine strategies.

## 9. Conclusions

Oxidative stress has emerged as an important biological component of IBD and is closely associated with immune activation, epithelial dysfunction, mitochondrial impairment, and disruption of redox homeostasis. Experimental and clinical studies support its involvement in multiple aspects of intestinal inflammation, although the precise contribution of oxidative stress to disease initiation and progression remains incompletely understood.

Growing evidence suggests that oxidative biomarkers and redox-sensitive molecular pathways may provide valuable insights into disease biology and could contribute to future biomarker-guided and precision medicine approaches. However, their current clinical application is limited by methodological heterogeneity, insufficient standardization, and the lack of robust prospective validation. At present, these biomarkers should be regarded primarily as investigational tools rather than routine diagnostic or prognostic markers.

Future research should focus on standardized biomarker assessment, longitudinal clinical validation, and integration of redox biology with molecular, microbiome, and multi-omics approaches. Such efforts may improve disease stratification, facilitate the development of targeted therapeutic strategies, and further clarify the role of oxidative stress within the complex pathophysiology of IBD.

## Figures and Tables

**Table 1:** Representative Studies Investigating Oxidative Stress Pathways, Biomarkers, and Translational Implications in Inflammatory Bowel Disease.

First Author	Study Design	Oxidative Biomarker/ Pathway	Main Mechanistic Finding	Translational Relevance	Major Limitation	Disease Context
Curran et al. (1991) [11]	Comparative experimental study	Superoxide anion production	Demonstrated increased neutrophil-derived ROS production associated with intestinal inflammation	Supports ROS-mediated inflammatory amplification in active IBD	Early observational mechanistic study	Crohn’s disease
Baldassano et al. (1993) [12]	Comparative study	Superoxide production	Reported enhanced monocyte oxidative burst during inflammatory activation	Suggests contribution of innate immune oxidative activation in IBD pathophysiology	Limited mechanistic specificity	Crohn’s disease
Roediger et al. (1986) [13]	Cross-sectional observational study	Nitrite concentrations	Identified elevated colonic nitrite levels in active mucosal inflammation	Supports involvement of nitrosative stress pathways in ulcerative colitis	Cross-sectional design without longitudinal validation	Ulcerative colitis
Perner et al. (1999) [14]	Review study	Nitric oxide and peroxynitrite signaling	Highlighted mechanistic role of NO-related pathways in chronic intestinal inflammation	Supports translational relevance of nitrosative signaling in IBD progression	Narrative review without standardized biomarker assessment	Crohn’s disease, Ulcerative colitis
Oudkerk Pool et al. (1995) [15]	Cross-sectional comparative study	Serum nitrate levels	Reported increased circulating nitrate concentrations during active inflammation	Suggests potential systemic oxidative biomarkers associated with disease activity	Systemic nitrate variability and limited specificity	Crohn’s disease, Ulcerative colitis
Hong et al. (2010) [21]	Cross-sectional study	Spermine oxidase (SMOX) expression	Demonstrated increased SMOX expression associated with oxidative epithelial injury	Identifies polyamine oxidative pathways as potential translational targets	Limited patient stratification	Ulcerative colitis
Yamamoto et al. (2015) [25]	Prospective observational study	Oxidative stress ratio after anti-TNF therapy	Demonstrated reduction in oxidative stress following biologic therapy	Supports therapeutic modulation of oxidative pathways during anti-TNF treatment	Limited longitudinal follow-up	Crohn’s disease
Passos et al. (2023) [7]	Systematic review	Serum thiol concentrations	Reported decreased thiol levels reflecting impaired antioxidant defenses	Supports translational relevance of antioxidant depletion biomarkers	Biomarker heterogeneity across included studies	Crohn’s disease, Ulcerative colitis
Tratenšek et al. (2024) [8]	Systematic review	Oxidative and antioxidant biomarkers	Systematically evaluated oxidative stress-related biomarkers associated with IBD activity	Supports biomarker-guided assessment of inflammatory activity	Methodological variability among included studies	Crohn’s disease, Ulcerative colitis
Xu et al. (2023) [23]	Multi-omics Mendelian randomization analysis	Oxidative stress-related genes and microbiome interactions	Identified oxidative stress gene signatures linked to microbiome dysregulation	Supports molecular stratification and redox-associated endotypes	Limited direct clinical validation	Crohn’s disease
van Asseldonk et al. (2024) [26]	Randomized pilot trial	Oxidative biomarkers during thiopurine therapy	Evaluated oxidative biomarker modulation during therapeutic intervention	Suggests utility of oxidative biomarkers for therapeutic monitoring	Small pilot study population	Crohn’s disease, Ulcerative colitis
Narimani et al. (2024) [27]	Clinical dietary intervention study	Oxidative biomarkers and TAC	Demonstrated improvement in antioxidant capacity following Mediterranean dietary intervention	Supports dietary modulation of oxidative stress pathways	Short-term interventional assessment	Ulcerative colitis

Abbreviations: IBD, inflammatory bowel disease; NO, nitric oxide; ROS, reactive oxygen species; SMOX, spermine oxidase; TAC, total antioxidant capacity; TNF, tumor necrosis factor.

**Table 2:** Oxidative Stress and Antioxidant Biomarkers Associated with Inflammatory Bowel Disease Activity and Translational Relevance.

Biomarker	Mechanistic Role	Pattern in Active Disease	Translational Implication	Major Limitation	Key References
Nitric oxide (NO) and nitrite/nitrate metabolites	Nitrosative signaling and iNOS-mediated inflammatory activation	↑ Increased in active ulcerative colitis and Crohn’s disease	Reflects nitrosative inflammatory burden and mucosal inflammatory activity	Influenced by dietary nitrate intake, systemic metabolism, and assay variability	[13,14,15,16,28]
Malondialdehyde (MDA)	Lipid peroxidation and oxidative membrane injury	↑ Increased in active disease	Indicates oxidative tissue injury and lipid oxidative damage	Limited specificity for intestinal inflammation	[4,6,19,27,28]
Myeloperoxidase (MPO)	Neutrophil-derived oxidative enzymatic activity	↑ Increased in active disease	Reflects neutrophil-mediated oxidative inflammatory activation	Elevated in multiple systemic inflammatory conditions	[4,6,27,28]
Superoxide anion (O_2_^−^)	Reactive oxygen species generation during oxidative burst activation	↑ Increased in active disease	Supports innate immune-cell oxidative activation and inflammatory amplification	Primarily experimental and difficult to standardize clinically	[4,6,11,12]
Total antioxidant capacity (TAC)	Global systemic antioxidant defense reserve	↓ Reduced in active disease	Reflects overall antioxidant depletion during inflammatory activity	Heterogeneous analytical methodologies and poor inter-study standardization	[5,6,28]
Thiol groups (–SH)	Non-enzymatic redox buffering and glutathione-associated antioxidant defense	↓ Reduced in active disease	Indicates impaired systemic redox buffering capacity and antioxidant reserve depletion	Influenced by nutritional and metabolic variability	[7,20]
Antioxidant enzymes (GPx, SOD, CAT)	Enzymatic detoxification of reactive oxygen intermediates and peroxide metabolism	↓ Reduced in active ulcerative colitis and Crohn’s disease	Suggests impaired enzymatic antioxidant defense and oxidative stress persistence	Variable tissue distribution and assay heterogeneity	[7,8,18,20]
Oxidative stress-related genes (NOX1, DUOX2, PRDX1, SMOX)	Molecular regulation of ROS generation, epithelial oxidative signaling, and inflammatory modulation	Dysregulated expression in active disease	Supports molecular stratification and redox-associated inflammatory endotypes	Limited direct clinical validation and absence of standardized molecular integration	[21,23]
Ferroptosis-associated pathways (GPX4/glutathione depletion)	Iron-dependent lipid peroxidation and oxidative epithelial cell death	Emerging evidence of activation during inflammatory oxidative injury	Potential future target for redox-modulating therapeutic strategies	Primarily experimental evidence with limited translational validation	[24]

Abbreviations: CAT, catalase; DUOX2, dual oxidase 2; GPX4, glutathione peroxidase 4; GPx, glutathione peroxidase; iNOS, inducible nitric oxide synthase; MDA, malondialdehyde; MPO, myeloperoxidase; NO, nitric oxide; NOX1, NADPH oxidase 1; PRDX1, peroxiredoxin 1; ROS, reactive oxygen species; SMOX, spermine oxidase; SOD, superoxide dismutase; TAC, total antioxidant capacity; ↑, increased; ↓, decreased.

## Data Availability

No new data were created or analyzed in this study. Data sharing is not applicable to this article.

## Abbreviations

CAT	Catalase
DUOX2	Dual Oxidase 2
GPx	Glutathione Peroxidase
GPX4	Glutathione Peroxidase 4
IBD	Inflammatory Bowel Disease
iNOS	Inducible Nitric Oxide Synthase
MDA	Malondialdehyde
MPO	Myeloperoxidase
NAC	N-acetylcysteine
NF-κB	Nuclear Factor kappa B
NO	Nitric Oxide
NOX1	NADPH Oxidase 1
Nrf2	Nuclear Factor Erythroid 2-Related Factor 2
PRDX1	Peroxiredoxin 1
RNS	Reactive Nitrogen Species
ROS	Reactive Oxygen Species
SMOX	Spermine Oxidase
SOD	Superoxide Dismutase
TAC	Total Antioxidant Capacity
AUC	Area Under the Receiver Operating Characteristic Curve
ATP	Adenosine Triphosphate
CRP	C-Reactive Protein
ESR	Erythrocyte Sedimentation Rate
MeSH	Medical Subject Headings
UC	Ulcerative Colitis
CD	Crohn’s Disease
